# Orchestration of Tumor-Associated Macrophages in the Tumor Cell-Macrophage-CD8^+^ T Cell Loop for Cancer Immunotherapy

**DOI:** 10.7150/ijbs.115932

**Published:** 2025-06-12

**Authors:** Lin He, Paul Kwong-Hang Tam, Chu-Xia Deng

**Affiliations:** 1 Cancer Center, Faculty of Health Sciences, University of Macau, Macau SAR, China.; 2 Centre for Precision Medicine Research and Training, Faculty of Health Sciences, University of Macau, Macau SAR, China.; 3 MOE Frontier Science Centre for Precision Oncology, University of Macau, Macau SAR, China.; 4 Medical Sciences Division, Macau University of Science and Technology, Macau SAR, China.; 5 Precision Regenerative Medicine Research Centre, Macau University of Science and Technology, Macau SAR, China.

**Keywords:** tumor-associated macrophage, CD8^+^ T cell, tumor microenvironment, cancer immunotherapy

## Abstract

The tumor microenvironment is densely populated with tumor-associated macrophages (TAMs), which exhibit various phenotypes at different stages of tumor progression. TAMs are highly plastic and intricately linked to the antitumor activity and functionality of CD8^+^ T cells. Tumor cells, TAMs and CD8^+^ T cells constitute a feedback loop that monitors the tumor immune surveillance. Modulation of several chief signaling pathways within TAMs can steer them towards either an immunoinflammatory or immunosuppressive state. This can be achieved indirectly through cancer therapies or by directly targeting TAMs. New detailed insights into the immunostimulatory reprogramming of TAMs inspire the design of novel combinatory strategies that can be extrapolated to bolster cancer immunotherapy.

## 1. Introduction

**Figure 1:**
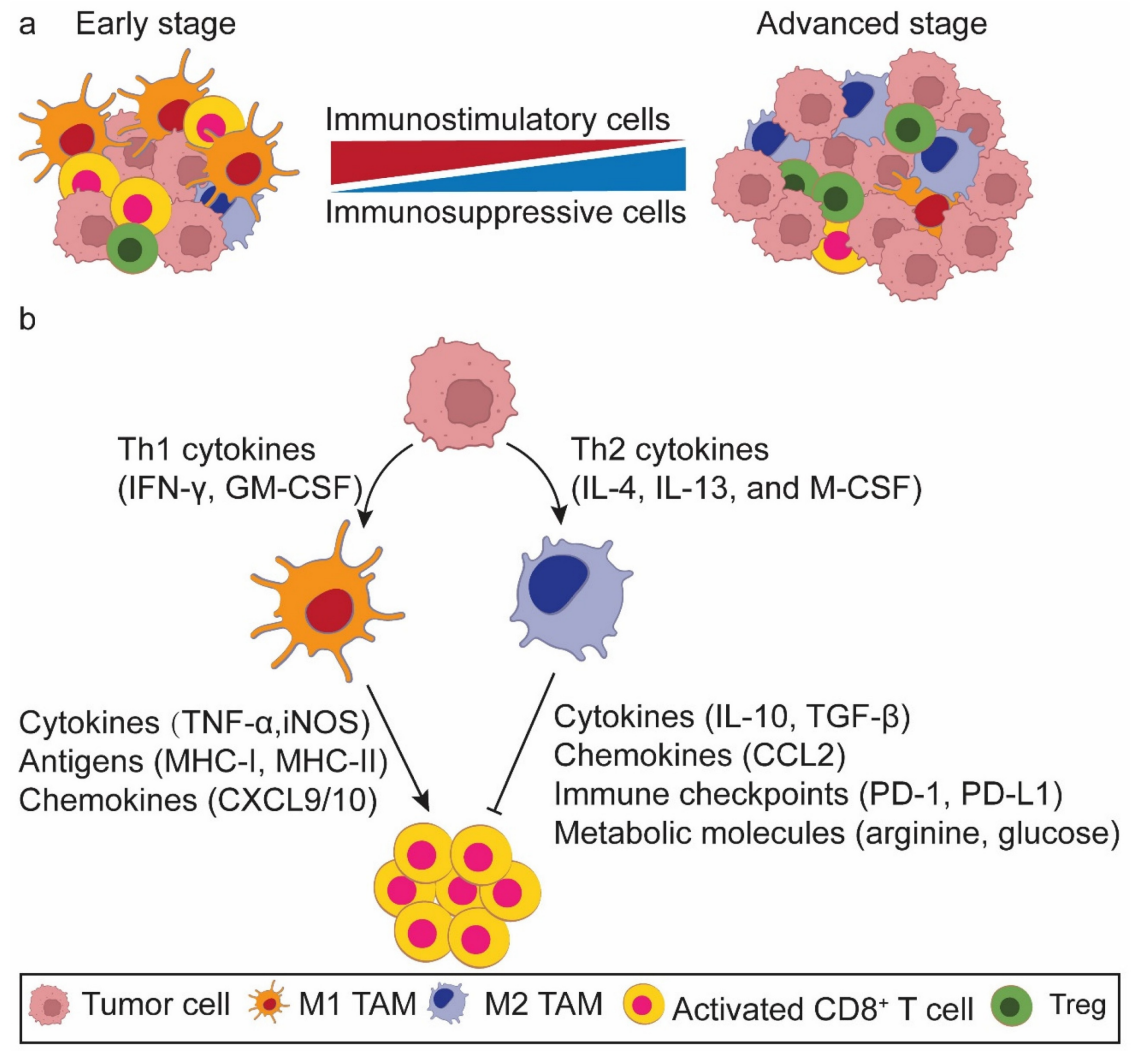
** A conceptual model of the tumor cell-macrophage-CD8^+^ T cell loop in the tumor microenvironment (TME). a. Alterations of macrophage phenotype and CD8^+^ T cell functionality in the TME during tumorigenesis.** In the early stage of tumorigenesis, the TME is predominantly populated by M1 tumor associated macrophages (TAMs) and activated CD8^+^ T cells. Conversely, in the advanced stage, M2 TAMs and exhausted CD8^+^ T cells pervade the TME. **b. Macrophages act as the bridge to orchestrates the interaction of tumor cells and CD8^+^ T cells.** Tumor cells secrete an array of cytokines to educate the polarization state of TAMs. These TAMs, in turn, change the antitumor efficacy of CD8^+^ T cells via multiple mechanisms.

Immune cells are generally classified into innate and adaptive immune cells, with macrophages and T cells mapping to main representatives of each category, respectively. Macrophages serve as the body's first line of defense against pathogens and cancers^1^. They directly eliminate senescent cells and tumor cells through phagocytosis or indirectly kill them via revitalizing CD8^+^ T cells. The phagocytosis of macrophages is reliant on “eat me” and “don't eat me” signals^2^. Tumor cells frequently overexpress CD47, which, upon binding to SIRPα on the surface of macrophages, transmits a “don't eat me” signal. This interaction between CD47 and SIRPα attenuates the phagocytic activity of macrophages, enabling tumor cells to obviate destruction^3^. As such, macrophages further partake in both innate and adaptive immunity^4^. These immune cells play drastically distinct roles at different stages of tumor progression by altering their phenotypes. In the early stage a high proportion of proinflammatory tumor-associated macrophages (M1 TAMs) and activated CD8^+^ T cells accumulate to remove the initially formed tumor cells. Conversely, in the late stage the TME is characterized by an increasing body of immunosuppressive TAMs (M2 TAMs) and exhausted CD8^+^ T cells, which promote tumor progression and metastasis. M1 TAMs potentiate T cell responses, whereas M2 TAMs attenuate T cell function and cancer immune surveillance within the TME **(Figure 1a)**. Therefore, TAMs play dual roles in immune activation and suppression^5^, ^6^. It is important to note that macrophage phenotypes exist along as a dynamic continuum rather than as rigid binary classification. This spectrum reflects their functional plasticity in response to microenvironmental cues.

Macrophages are highly plastic immune cells within the TME^7^, ^8^. Lipopolysaccharide (LPS), either alone or in combination with Th1 cytokines such as interferon-γ (IFN-γ) and granulocyte-macrophage colony-stimulating factor (GM-CSF), facilitates the polarization of TAMs towards the M1 phenotype. This polarization is characterized by the production of proinflammatory cytokines [interlukin-6 (IL-6), IFN-γ, tumor necrosis factor-α (TNFα), and inducible nitric oxide synthase (iNOS)], augmented antigen presentation, and heightened phagocytic activity^9^. Conversely, Th2 cytokines such as IL-4, IL-13, and macrophage colony-stimulating factor (M-CSF) reprogram TAMs towards the M2 phenotype, leading to the secretion of anti-inflammatory cytokines like IL-10 and transforming growth factor-β (TGF-β), and elevated expression of CD163, CD206, and Arg-1^9^.

Currently, it is envisaged that TAMs modulate the CD8^+^ T cell function through five principal underpinnings **(Figure 1b)**: (1) cytokine secretion^10^, ^11^, (2) antigen presentation^12^, (3) chemokine production to recruit CD8^+^ T cells^13^, (4) immune checkpoint modulation [e.g., programmed death receptor 1 (PD-1), programmed death ligand 1 (PD-L1), and B7-H3 (CD276)]^12^, ^14^, ^15^, and (5) metabolism modulation (e.g., arginine, glucose, and lactate)^14^-^16^. Ideally, TAMs and activated CD8^+^ T cells work synergistically to identify and eliminate primary and metastatic tumor cells, potentially maximizing the tumoricidal potency.

Interactions between tumor cells and immune cells play a crucial role in controlling tumorigenesis. Reprogramming immune cells within the TME may enhance the efficacy of cancer immunotherapy. In this review, we propose a tumor cell-macrophage-CD8^+^ T cell loop by wiring a connection between TAMs and CD8^+^ T cells across multiple tumor landscapes. Several crucial signaling pathways within TAMs that regulate their immunostimulatory or immunosuppressive polarization are further discussed. Orchestration of these signaling pathways can yield a myriad of treatment scenarios to overcome TAM-mediated immunosuppressive TME. Finally, we focus on strategies to immunostimulate the accumulation and functionality of TAMs and CD8^+^ T cells within the TME, exploring potential therapeutic avenues for combining immune checkpoint blockade (ICB) therapy.

## 2. Tumor Cell-Macrophage-CD8^+^ T Cell Loop

Tumors with diverse gene expression demonstrate significant correlations with the congregation of distinct phenotypes of TAMs and CD8^+^ T cells **(Table 1)**. Elevated expression of MICAL2 in pancreatic cancer is associated with a heightened infiltration of M2 TAMs and a diminished presence of CD8^+^ T cells^17^. YBX1+ luminal breast cancer tissues demonstrate substantial infiltration of M2 TAMs and elevated expression of T cell exhaustion markers, such as indoleamine 2,3-dioxygenase 1 and cytotoxic T-lymphocyte-associated protein 4 (CTLA4)^18^. In patients with intrahepatic cholangiocarcinoma, GM-CSFRα is positively correlated with CD8^+^ T cell infiltration, while inversely associated with the presence of M2 TAMs and myeloid-derived suppressor cells (MDSCs)^19^. KCTD5 shows a positive correlation with the accumulation of CD8^+^ T cells and TAMs in patients with lung adenocarcinoma (LUAD)^20^. Breast cancers with overexpression of ARHGAP39 exhibit low infiltration levels of CD8^+^ T cells and macrophages, but high infiltration of CD4^+^ T cells^21^. OPN deficiency in glioma cells reduces M2 TAM populations and increases CD8^+^ T cell cytotoxicity^22^. MTA1-overexpressing colon cancer weakens CD8^+^ T cell responses by inducing TAMs^23^. In hepatocellular carcinoma (HCC) tissues with high MISP expression, M2 TAMs are scarce, while CD8^+^ T cells are diffusely infiltrated^24^. GPC3-overexpressing ovarian tumor enhances M1 TAM infiltration and triggers a specific CD8^+^ T cell response, thereby improving the long-term survival of mice^25^.

Given the proinflammatory and anti-inflammatory characteristics, specific molecular biomarkers for TAMs have been delineated through advanced molecular biology techniques. These newly characterized TAMs exhibit a robust correlation with the presence of CD8^+^ T cells in different tumor types. TIM4^+^ macrophages are predominantly located within the T-cell zones of tertiary lymphoid structures associated with various malignancies, showing a positive correlation with CD8^+^ T cell infiltration^26^. CD163^+^ M2 TAMs accumulate within the stromal compartments at the tumor-stroma interface of clear cell renal cell carcinoma and are positively correlated with an increased proportion of TIM3^+^CD8^+^ T cells, indicating terminal exhaustion^27^. JMJD8^+^ M2 TAMs exhibit a positive correlation with the presence of immunosuppressive cells and the suppression of CD8^+^ T cell function across various cancer types^28^.

Cumulatively, modulating the expression levels of specific genes in tumor cells can profoundly influence the TAM phenotype and CD8^+^ T cell antitumor immunity. Likewise, genetic alterations in TAMs closely changes CD8^+^ T cell presence and activation within the TME. Tumor cells, macrophages, and CD8^+^ T cells constitute a feedback loop that orchestrates tumor immune surveillance; wherein, TAMs are pivotal determinants. Consequently, the phenotypic modulation of TAMs is critical for either reversing or propelling tumor immune evasion.

Recent studies have elucidated valuable insights into the temporal and spatial heterogeneity of TAMs and their interactions with CD8^+^ T cells, which are pivotal for comprehending the cancer-TAM-CD8^+^ T cell loop and its role in tumor immunity. In the early stages of tumor development, the TME is characterized by the presence of M1 TAMs, activated CD8^+^ T cells, and proinflammatory cytokines, which can aid in controlling tumor growth^29^. As tumors progress, the TME undergoes profound alterations, leading to an immunosuppressive state. This shift is marked by the increased presence of regulatory T cells (Tregs), MDSCs, M2 TAMs, and immunosuppressive cytokines. These changes inhibit the function of CD8^+^ T cells and promote tumor growth and metastasis^29^, ^30^.

The spatial distribution of TAMs within the TME can significantly modulate their interactions with CD8^+^ T cells. For example, TAMs located near blood vessels may have different functional properties compared to those in hypoxic regions of the tumor. These regional disparities can influence the recruitment, activation, and suppression of T cells^31^. Advanced techniques, such as single-cell RNA sequencing, have revealed the diversity of TAM subtypes and their distinct roles in various tumor regions. These studies have shown that TAMs can either potentiate or inhibit T cell function depending on their location and the specific signals they receive from the TME^32^. Reciprocally, immunotherapy-activated CD8^+^ T cells recruit TAMs to their vicinity via CCR5 signaling and educates them into M1 phenotypes. Therefore, effective immunotherapy requires coordinated functional modulation of both CD8^+^ T cells and TAMs^33^. Activated CD8^+^ T cells also secret cytokines to promote the M1 TAM polarization. Understanding the temporal and spatial heterogeneity of TAMs and their interactions with CD8^+^ T cells is essential for the development of effective cancer therapies.

## 3. M1 TAM Polarization Signaling Pathways

### 3.1. NF-κB signaling activation

**Figure 2:**
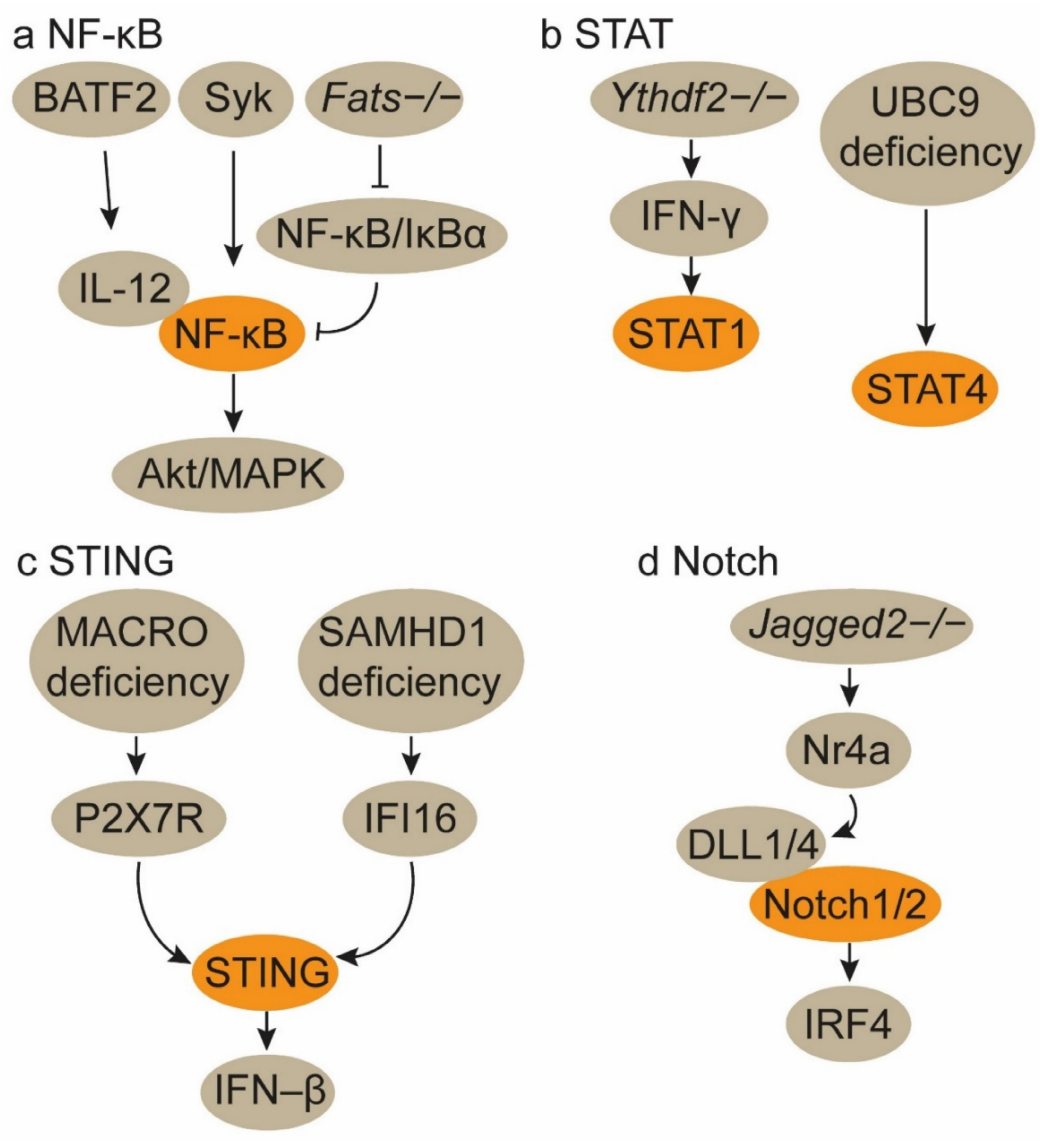
** Signaling pathways for the polarization of TAMs towards the M1 phenotype. a. Activation of the NF-κB signaling pathway. b. Activation of the STAT1/4 signaling pathways.** The activation of the NF-κB and STAT1/4 signaling pathways in TAMs induces their polarization towards the M1 phenotype. **c. Activation of the STING signaling pathway. MACRO deficiency in TAMs promotes M1 polarization by activating the P2X7R-mediated STING-IFN-β pathway.** Concurrently, the genetic deletion of SAMHD1 in tumor cells enhances the activation of the FI16-STING pathway, which subsequently polarizes TAMs to the M1 phenotype.** d. Activation of the Notch pathway. Jagged 2-/- lung tumor cells trigger Nr4a-mediated induction of the Notch ligands DLL1/4 in cancer cells.** DLL1/4 activates Notch1/2 signaling in macrophages, leading to M1 TAM polarization.

The basic leucine zipper transcription factor ATF-like 2 (*Batf2*) triggers CD8^+^ T cell antitumor immunity by upregulating IL-12 p40 in TAMs. Mechanistically, BATF2 interacts with p50 and p65 in macrophages, upregulating their activity in the nucleus. This interaction promotes IL-12 p40 expression in macrophages via nuclear factor kappa-B (NF-κB) binding site in the Il12b promoter^34^. ApoC3TG mice-derived macrophages, when co-cultured with CD8^+^ T cells, significantly enhance CD8^+^ T cell activation. ApoC3 binds to Toll-like receptor 2/4 (TLR2/TLR4), inducing the spleen tyrosine kinase (Syk) activation, which promotes inflammasome activation in macrophages through the NF-κB/Akt/MAPK and cPLA2/NOX2 pathways^35^. *Fats*-/- mice experience a shift of TAMs from an M2 to an M1 phenotype. This polarization is mediated by the stimulation and prolonged activation of NF-κB through the disruption of NF-κB/IκBα negative feedback loops, thereby enhancing the adaptive immune response of CD8^+^ T cells^36^. Morus alba L. fruit extract enhances the secretion of inflammatory cytokines and promotes M1 polarization in macrophages through the TLR4 downstream MAPKinase and NF-κB signaling pathways. This, in turn, amplifies CD8^+^ T cell activity and IFN-γ production^37^. An H2 receptor antagonist Ranitidine may activate the phosphoinositide 3-kinase (PI3K)-Akt2 signal, which subsequently regulates the NF-κB and GSK3β/Dynamin1 pathways to promote TAM polarization to the M1 phenotype and enhance macrophage endocytosis **(Figure 2a)**^38^. These findings collectively demonstrate that upregulation of NF-κB signaling pathway favors M1 TAM polarization and augments TAM-specific CD8^+^ T cell antitumor immunity **(Table 2)**.

However, in certain instances, the inactivation of the NF-κB signaling pathway is conducive to M1 TAM polarization and CD8^+^ T cell-mediated antitumor responses. In glioblastoma multiforme (GBM) mouse models, *NF-κB p65* knockout significantly increases M1 TAMs and CD8^+^ T cells, while reducing the populations of M2 TAMs and MDSCs^39^. In vitro co-culture models demonstrate that abrogating myeloid cell-associated NF-κB signaling enhances T cell proliferation and activation, as well as educates M2 to M1 polarization^39^.

### 3.2. STAT1/4 signaling activation

The signal transducer and activator of transcription (STAT) family of transcription factors plays distinct roles in modulating TAMs and CD8^+^ T cells within the TME. Activation of the STAT1 and STAT4 pathways orchestrates TAMs towards M1 phenotypes **(Table 2)**. The loss of *Ythdf2* in TAMs reprograms them towards an antitumor phenotype and enhances their antigen presentation crosstalk to CD8^+^ T cells by upregulating IFN-γ-STAT1 signaling, thereby increasing CD8^+^ T cell responses **(Figure 2b)**^40^. C1q^+^ macrophages express a repertoire of immunomodulatory ligands via METTL14-YTHDF2 axis-mediated N6-methyladenosine (m6A) methylation on Ebi3, thereby preserving the functionality of CD8^+^ T cells. Consequently, *Mettl14* or *Ythdf2* deficiency in C1q^+^ macrophages thwarts cytotoxic CD8^+^ T cell infiltration and facilitates the accumulation of dysfunctional CD8^+^ T cells^41^.

UBC9 in TAMs impedes their polarization towards the M1 phenotype by facilitating STAT4 SUMOylation. The targeted ablation of UBC9 in TAMs promotes M1 polarization and augments TAM-CD8^+^ T cell interactions, thereby amplifying CD8^+^ T cell responses **(Figure 2b)**^42^.

### 3.3. STING signaling activation

MACRO inhibits type I IFN secretion and antigen presentation in TAMs. Mechanistically, MACRO diminishes the accumulation of tumor-derived cGAMP and ATP in the TME, thereby impeding P2X7R-mediated activation of the stimulator of interferon genes (STING)-IFN-β pathway. Utilizing anti-MACRO neutralizing antibodies can restore the phagocytic activity and antigen presentation capabilities of TAMs, leading to increased CD8^+^ T cell infiltration^43^. However, SAMHD1 deficiency in tumor cells triggers type I IFN production via the activation of the cytosolic IFI16-STING pathway, concurrently fostering the polarization of TAMs towards the M1 phenotype and augmenting CD8^+^ T cell accumulation **(Figure 2c)**^44^. Therefore, STING signaling activation in macrophages and cancer cells both primes type I IFN secretion and M1 TAM polarization** (Table 2)**. RON expression in breast cancer tissues, which inhibits IRAK4-mediated type I IFN production, gives rise to sparse infiltration of macrophages and CD8^+^ T cells^45^.

### 3.4. Notch1/2 signaling activation

Jagged1-expressing tumor cells activate the Notch signaling pathway, culminating in the secretion of pluralistic cytokines that facilitate TAM recruitment. These TAMs subsequently attenuate the proliferation and cytotoxicity of CD8^+^ T cells. Consequently, in triple-negative breast cancer models with elevated Jagged1 expression, TAM infiltration is pronounced while CD8^+^ T cell presence is markedly assuaged^46^. In non-small cell lung carcinoma models, the absence of Jagged2, rather than Jagged1, shields CD8^+^ T cell functionality. Jagged2-deficient lung cancers exhibit increased infiltration of M1 TAMs and CD8^+^ T cells. Mechanistically, deletion of *Jagged2* triggers *Nr4a*-mediated induction of the Notch ligands DLL1/4 in cancer cells. DLL1/4 activates Notch1/2 signaling in macrophages, inducing the expression of the transcription factor IRF4 to sustain their immunostimulatory phenotype **(Figure 2d)**^47^. These findings suggest that Jagged1 and Jagged2 respectively modulate the Notch pathway in tumor cells and macrophages to maintain an immunosuppressive TME mediated by macrophages and CD8^+^ T cells **(Table 2)**.

### 3.5. Pattern recognition receptor signaling activation

Pattern recognition receptors (PRRs), including TLRs, Nod-like receptors (NLRs), C-type lectin receptors (CLRs), and RIG-I-like receptors (RLRs), recognize specific pathogen-associated molecular patterns (PAMPs) and damage-associated molecular patterns (DAMPs) and play dual roles in macrophage polarization and immunomodulation^48^. PRRs orchestrate the immune response by regulating the production of cytokines and chemokines. M1 TAMs are induced by PRR signaling in response to microbial products and proinflammatory cytokines, leading to the production of inflammatory mediators like TNF-α and IL-6. Conversely, M2 TAMs are promoted by PRR signaling in response to anti-inflammatory signals, resulting in the production of anti-inflammatory cytokines like IL-10 and TGF-β^48^, ^49^. These regulatory mechanisms facilitate the recruitment and activation of other immune cells, such as T cells and dendritic cells, thereby shaping the overall immune response^49^. In the context of cancer, PRRs on TAMs can either promote or inhibit tumor progression. For example, PRR activation can lead to the production of cytokines that enhance antitumor immunity or, alternatively, create an immunosuppressive environment that supports tumor growth^50^. The role of PRRs in macrophages is crucial for developing targeted therapies that can modulate macrophage function in various diseases, including cancer.

## 4. M2 TAM Polarization Signaling Pathways

TAMs primarily appear as an M2 phenotype within the TME at late stages. This phenomenon is largely by virtue of the myriad of materials produced during tumor progression, which, upon embedding into macrophages, alter the activation states of several critical signaling pathways **(Table 2)**. In turn, these M2 TAMs secrete extracellular vesicles and cytokines that foster tumor immune evasion.

### 4.1. NF-κB signaling inactivation

**Figure 3:**
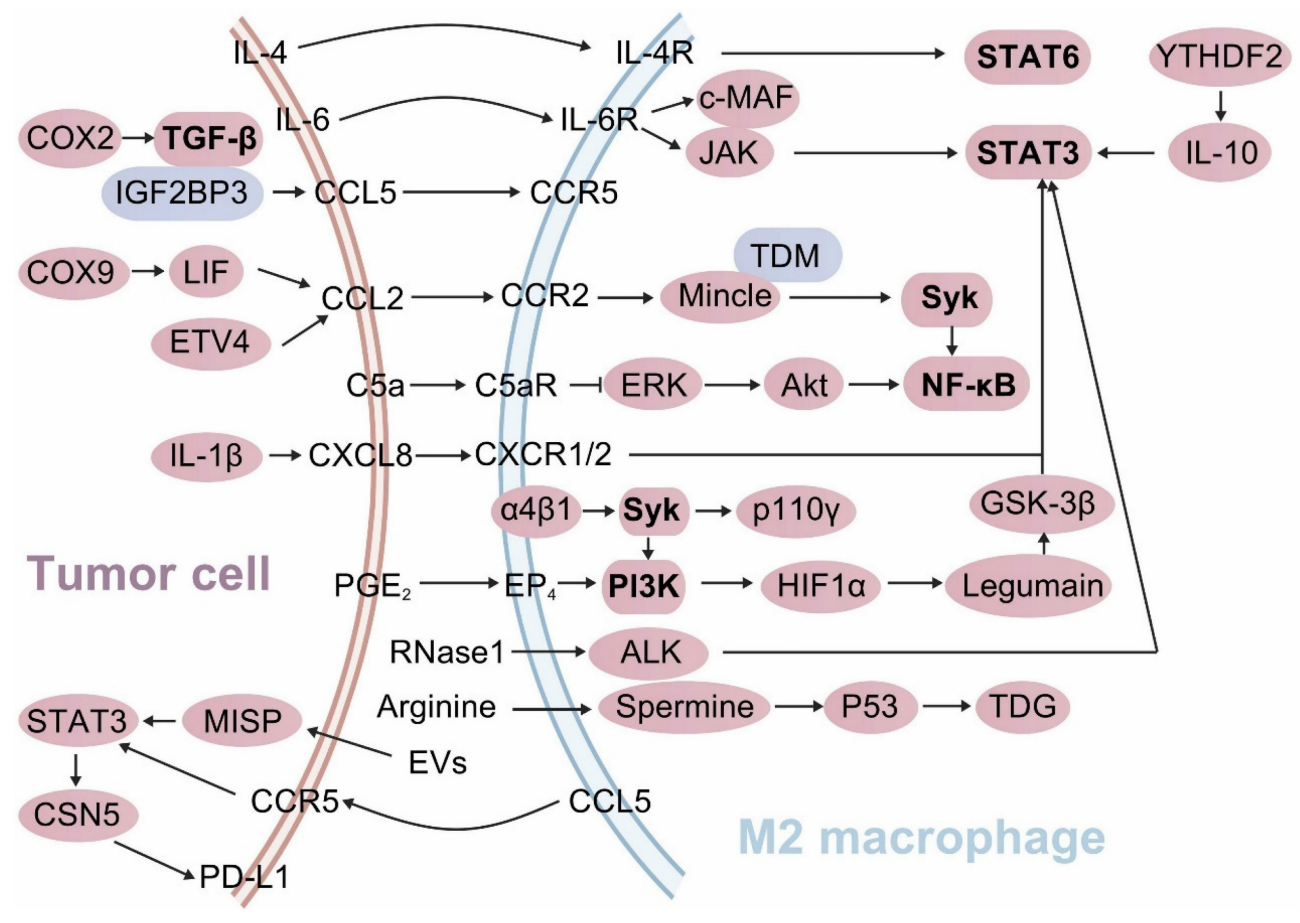
** Tumor cell-macrophage interactions reprogram M2 TAM polarization.** Tumor cells release a panel of chemokines, cytokines, PEG_2_, RNase1, and arginine to educate macrophages to M2 phenotypes. These molecules engulfed by macrophages induce their immunosuppressive polarization by activating STAT3/6, Syk-PI3K signaling pathways and suppressing NF-κB signaling activity. Moreover, tumor cell-derived arginine orchestrates macrophages towards M2 TAMs via activating arginine-polyamine- thymine DNA glycosylase (TDG) axis. In turn, M2 TAMs secrete extracellular vesicles (EVs) and chemokines (blue color) into tumor cells, which upregulate their PD-L1 expression by activating the STAT3 signaling pathway, thereby facilitating tumor immune evasion.

The complement component 5a-complement component 5a receptor (C5a-C5aR) axis modulates macrophage function and antitumor immunity. C5a facilitates M2 TAM infiltration and tumor cell metastasis. Conversely, C5aR deficiency reinstates TAM antitumor activity and augments TAM-mediated CD8^+^ T cell responses. Mechanistically, the C5a-C5aR axis suppresses macrophage C-X-C motif chemokine ligand 9 (CXCL9) secretion by activating C/EBPβ and inhibiting the ERK/Akt/NF-κB signaling pathways^51^. In macrophages, annexin A1 (ANXA1) promotes TAM polarization towards the M2 phenotype by downregulating the NF-κB and Notch1 pathways, while upregulating the JAK-STAT, Akt, and ERK pathways. ANXA1 deficiency increases the M1/M2 ratio and augments CD8^+^ T cell activation **(Figure 3)**^52^. Phospholipase A2 Group VII (PLA2G7) promotes the polarization of TAMs towards the M2 phenotype in HCC candidates by downregulating the NF-κB pathway, thereby suppressing CD8^+^ T cell responses^53^.

The cyclooxygenase-2 (COX-2)-derived oncogenic promoter PGE_2_ augments the affinity of NF-κB to the PD-1 promoter in both macrophages and CD8^+^ T cells via the EP4-PI3K-Akt signaling cascade, thereby upregulating their PD-1 expression. Conversely, the EP4-PI3K-Akt signaling blockade enhances macrophage phagocytosis and CD8^+^ T cell proliferation and activation in CRC models^54^. However, in SIGLEC10^+^ macrophages, Akt/P38/Erk signaling activity is suppressed, which educates their polarization towards the M2 phenotype and mitigates the CD8^+^ T cell proliferation and activation **(Figure 3)**^55^. Akt phosphorylates MAPK-activating death domain protein, which subsequently activates Rab27a, leading to enhanced secretion of PD-L1-enriched exosomes from TAMs^56^.

### 4.2. STAT3/6 signaling activation

Activation of the STAT3 and STAT6 signaling pathways induces M2 phenotype polarization and fosters an immunosuppressive TME. The STAT3 signaling pathway is crucial for maintaining the M2 phenotype of TAMs across various tumor models. Tumor cell-derived RNase1 induces the polarization of TAMs from the M1 to the M2 phenotype by activating ALK/STAT3 signaling and attenuating STAT1 phosphorylation^57^. In macrophages, the m6A reader YTHDF2 educates an anti-inflammatory phenotype by upregulating IL-10-STAT3 signaling^40^. Hypoxia inducible factor 1 α (HIF1α) transcriptionally upregulates Legumain in TAMs, which subsequently induces M2 polarization by activating the GSK-3β-STAT3 signaling pathway **(Figure 3)**. Disrupting the HIF1α-Legumain axis can attenuate M2 TAM polarization and potentiate CD8^+^ T cell antitumor immunity^58^. YAP and STAT3 are overactivated and form a complex in breast cancers. Inhibition of the YAP/STAT3 complex induces M1 TAM polarization, curtails Tregs populations, and amplifies CD8^+^ T cell activity^59^. Progranulin markedly upregulates PD-L1 expression on macrophages and drives their polarization towards the M2 phenotype via the JAK/STAT3 signaling pathway. This process inhibits CD8^+^ T cell proliferation and activation through the PD-1/PD-L1 interaction. Conversely, this effect can be abrogated by the STAT3 inhibitor Stattic^60^.

Within the senescent TME, the senescence-associated secretory phenotype-associated proinflammatory cytokine IL-6 modulates CD73 expression in TAMs via the JAK/STAT3 signaling cascade **(Figure 3)**. This elevates adenosine levels and attenuates CD8^+^ T cell antitumor immunity^61^. The c-MAF and STAT3 signaling pathways are crucial for IL-6 to sustain the LYVE-1^+^ TAM phenotype. IL-6 induces LYVE-1^+^ TAMs to express the immunosuppressive enzyme heme oxygenase-1 and form LYVE-1^+^ TAM nests via a CCR5-dependent signaling axis, thereby suppressing CD8^+^ T cell recruitment^62^. However, early IL-6 signal blockade weakens M1 TAM functionality through reducing SOCS3 levels and increasing SIRP levels, and thus decreasing the CD8^+^ T cell responses^63^-^65^.

STAT6 is another regulator of the M2 TAM transcriptional program within the STAT family. In the nucleus, phosphorylated STAT6 induces the transcription of M2 signature genes such as *Arg1*, *Ccl17*, and *Mrc1*, while concurrently inhibiting the activation of M1 signature genes like *Nos2*, *Ccl5*, and *Nlrp3*^66^, ^67^. Multiple cells within the TME produce IL-4, which polarizes TAMs towards the M2 phenotype via activating the STAT6 pathway **(Figure 3)**^68^, ^69^. However, *Stat6*^-/-^ tumor-bearing mice-derived TAMs exhibit an M1 phenotype^70^. Plus, exoASO-STAT6 treatment increases the M1/M2 ratio and CD8^+^ T cell activation in mice, further substantiating the role of STAT6 in the M2 polarization^71^.

### 4.3. Mincle pathway activation

Tumor cells secrete various cytokines and chemokines that recruit and activate macrophages^72^. Once recruited to the TME, macrophages can express Mincle (Clec4e), a CLR that recognizes certain glycolipids (particularly trehalose dimycolate) and damaged cell components^73^. Upon activation, Mincle triggers downstream signaling pathways that modulate macrophage behavior^73^. Specifically, Mincle engages Syk to subsequently activates the NF-κB signaling pathway. This pathway is crucial for the transcription of genes associated with the M2 TAMs **(Figure 3)**^73^. The activation of the Mincle/Syk/NF-κB signaling axis promotes the expression of genes that are characteristic of M2 TAMs^73^, ^74^. Conversely, silencing *Mincle* has been shown to enhance the proinflammatory and antitumor activities of M1 TAMs^73^. This suggests that Mincle signaling actively suppresses the functionality of M1 TAMs while contributes to the polarization of M2 TAMs.

### 4.4. Syk-PI3K signaling activation

The activation of Syk-PI3K axis in macrophages drives their polarization towards the M2 phenotype, thereby establishing an immunosuppressive microenvironment in vivo. Pharmacological dual-targeting Syk and PI3K in macrophages can reprogram them towards the M1 phenotype. This intervention disrupts the α4β1-Syk-p110γ axis in macrophages, creating the destabilization of HIF1α and ultimately restoring the functionality of CD8^+^ T cells^75^. PI3Kγ signaling in macrophages leads to NF-κB inactivation and C/EBPβ activation via Akt/mTOR axis, thus initiating an immunosuppressive transcriptional program that undermines CD8^+^ T cell antitumor immunity **(Figure 3)**^76^. *Clever-1* deficiency in macrophages amplifies their immunostimulatory activity by enhancing mTOR signaling, thereby reactivating CD8^+^ T cell responses^77^.

Genetic deletion of *Syk* in macrophages educates them towards the M1 phenotype, therefore augmenting CD8^+^ T cell infiltration and activation. Similarly, the FDA-approved Syk inhibitor R788 induces TAM polarization towards the M1 phenotype and promotes CD8^+^ T cell activation in pancreatic ductal adenocarcinoma (PDAC) mice^78^. PI3Kγ is a marker of TAMs in PDAC and drives their polarization towards an immunosuppressive phenotype. Inhibition of PI3Kγ reprograms the transcriptional profile of TAMs, thereby activating CD8^+^ T cell-mediated immune surveillance^79^. In PDAC mice, B cell-macrophage crosstalk promotes TAM polarization towards the M2 phenotype via the activation of PI3Kγ/BTK axis **(Figure 3)**. Thus, PI3Kγ inhibitor or BTK inhibitor treatment induces macrophage polarization towards the M1 phenotype and restores CD8^+^ T cell cytotoxicity^80^. Therefore, the suppression of the Syk/PI3K axis activity in macrophages reverses their immunosuppressive phenotype and rescues the tumor immune evasion.

### 4.5. Enhanced crosstalk of chemokines and their receptors

TAMs facilitate PD-1 expression on CD8^+^ T cells through IRF8-dependent antigen presentation, thereby precipitating CD8^+^ T cell exhaustion^81^. IL-1β in the TME enhances CXCL8 secretion by tumor cells and the chemotaxis of M2 TAMs. Tumor-derived CXCL8 fosters M2 TAM polarization by activating the STAT3 signaling pathway and concurrently hurdles PD-1+ CD8^+^ T cell recruitment^82^. TAM-derived CCL5 facilitates immune evasion in colorectal cancer (CRC) through the p65/STAT3-CSN5-PD-L1 pathway^83^. Tumor cell-derived exosomes orchestrate the differentiation of monocytes into PD-1^+^ TAMs, which experience an M2-like phenotype with decreased phagocytic capacity and effectively suppress CD8^+^ T cell responses^84^. M2 TAM-derived extracellular vesicles enhance IQGAP1 nuclear translocation and activate STAT3 phosphorylation by downregulating MISP in HCC. This process attenuates CD8^+^ T cell responses and upregulates PD-L1 expression in tumor cells, thereby facilitating tumor immune evasion **(Figure 3)**^24^. Act1 downregulation in macrophages increases CXCL9/10 and PD-L1 expressions by activating the STAT3 signaling pathway. Additionally, anti-Act1 macrophages facilitate the benign-to-malignant transition in CRC cells via the CXCL9/10-CXC chemokine receptor 3 (CXCR3) axis and induce CD8^+^ T cell exhaustion through the PD-L1/PD-1 axis^85^.

Tumor cell-derived SOX9 orchestrates the polarization of TAMs towards the M2 phenotype through the paracrine secretion of leukemia inhibitory factor (LIF), thereby attenuating CD8^+^ T cell function. LIF is abundantly present in malignant ascites. The ablation of SOX9 can curtail the levels of M2 TAM-induced immunosuppressive cytokines, such as C-C motif chemokine ligand 2 (CCL2) and IL10, and reinvigorate CD8^+^ T cell responses^86^. Inhibiting the CCL2/C-C motif chemokine receptor 2 (CCR2) axis can reprogram TAM polarization towards the M2 phenotype and revitalize CD8^+^ T cell responses, thereby mitigating the immunosuppressive state^87^. ETV4 upregulation in tumor cells augments the recruitment of TAMs and MDSCs via the CCL2/CCR2 axis, while concurrently inhibiting CD8^+^ T cell accumulation **(Figure 3)**. Moreover, ETV4 propels HCC metastasis through an FGF19-ETV4-FGFR4 positive feedback loop^88^. In a triple-negative breast cancer model, CD8^+^ T cells induce PD-L1 expression in TAMs at the marginal TME through the CCL2/PD-L1 axis^89^. Innate αβ T cells (iαβTs) demonstrate tumor-protective properties by reprogramming immunogenic macrophages in a CCR5-dependent manner and inhibiting CD8^+^ T cell activation through PD-L1/PD-1 interactions^90^. Hedgehog (Hh) signaling in TAMs drives M2 polarization through the downstream transcription factor Gli1, which modulates Krüppel-like factor 4 (Klf4). *Klf4* deficiency in macrophages manifests as an M1 phenotype. Tumor cell-derived Hh ligand sonic hedgehog further amplifies M2 TAM polarization. Hh-induced M2 TAMs attenuate the production of CXCL9 and CXCL10, thereby impeding CD8^+^ T cell recruitment^91^. Nasopharyngeal carcinoma (NPC) cells secrete IFN-stimulated gene 15 (ISG15), which remodels macrophages into the M2 subtype by activating the LFA-1/SFK/CCL18 axis. These ISG15^+^ M2 TAMs significantly impair CD8^+^ T cell responses. Clinically, the presence of ISG15^+^ M2 TAMs is frequently correlated with poor prognosis in NPC patients^92^.

In advanced CRC patients, TAMs-derived IL8 alters CD8^+^ T cell function by downregulating TIM3 expression through the IL8-CXCR2 axis^93^. NLRP3 signaling in macrophages drives the differentiation of CD4^+^ T cells into Tregs and inhibits CD8^+^ T cell activation, processes dependent on IL-10^94^. *Emp3*-overexpressing macrophages produce elevated levels of TNF-α, which downregulates IL-2Rα expression on CD8^+^ T cells, thereby mitigating their proliferation and activation^95^.

### 4.6. TGF-β signaling upregulation and metabolic dysregulation in tumor cells

IGF2BP3, by binding to CCL5 or TGF-β1, orchestrates the polarization of TAMs towards the M2 phenotype, therefore suppressing CD8^+^ T cell functionality^96^. Elevated COX-2 expression in HCC drives TAM polarization towards the M2 phenotype and precipitates CD8^+^ T cell exhaustion via the TGF-β signaling pathway **(Figure 3)**^97^. Conversely, COX-2 deficiency in TAMs promotes their polarization towards the M1 phenotype, thereby augmenting CD8^+^ T cell activity and immune surveillance^98^.

Another critical mechanism involves the regulation of immune responses by metabolic enzymes and metabolites. In the tumor microenvironment (TME), breast cancer cells act as the primary source of arginine, which polarizes M2 TAMs and suppresses CD8^+^ T cell-mediated antitumor activity. Therapeutic targeting of the arginine-polyamine-thymine DNA glycosylase (TDG) axis between cancer cells and M2 TAMs significantly inhibits breast cancer growth **(Figure 3)**^99^. Pathways associated with immunometabolic circuits are increasingly recognized as critical regulators of TAM-T cell interaction. These pathways integrate metabolic reprogramming and immune signaling to shape the functional dynamics of the tumor microenvironment.

## 5. Post-Transcriptional Regulations

The 2024 Nobel Prize in Physiology or Medicine was awarded jointly to Dr. Victor Ambros and Dr. Gary Ruvkun for their seminal discovery of microRNAs (miRNAs) for their pivotal roles in post-transcriptional gene regulation. This monumental work has profoundly enhanced our understanding of gene expression and its implications for a plethora of diseases, including the polarization of TAMs and CD8^+^ T cell responses within the TME.

miR-155 is highly expressed in M1 TAMs^100^, ^101^ and plays a pivotal role in the polarization of TAMs towards the M1 phenotype^102^, ^103^. Additionally, miR-155 expression in T cells enhances TAM activation by inducing IFN-inducible genes. A triple combination of anti-PD-1/PD-L1/CTLA-4 ICB therapy significantly restores antitumor immunity in miR-155 T cell conditional knockout mice through the activation of both T cells and TAMs^104^. miR-506 reprograms M2 TAMs into M1 phenotypes by targeting STAT3 signaling, thus promoting CD8^+^ T cell infiltration and enhancing the sensitivity of PDAC patients to PD-1 ICB therapy^105^. Extracellular vesicles, replete with bioactive substances, act as essential mediators of intercellular molecular transport and communication^106^. Extracellular vesicle-derived miR-155-5p from LUAD cells reprograms TAMs to an immunostimulatory phenotype and enhances CD8^+^ T cell activation, thereby inhibiting immune escape in immunocompetent mice^107^.

However, some tumor cell-derived miRNAs engulfed by TAMs lead to an education of M2 phenotype. CRC cell-derived extracellular vesicles contain miR-21-5p and miR-200a, which, upon uptake by TAMs, induce M2 polarization and upregulate PD-L1 expression through the PTEN/Akt and SOCS1/STAT1 signaling pathways. This process leads to diminished CD8^+^ T cell responses and promotes tumor progression^108^. Endoplasmic reticulum (ER) stress in tumor cells facilitates immune escape in solid tumors by modulating the TME. ER-stressed HCC cell-derived exosomes harbor high levels of miR-23a-3p. Mechanistically, miR-23a-3p upregulates PD-L1 expression in macrophages via the PTEN/PI3K/Akt signaling pathway, thereby inhibiting T cell function and increasing T cell apoptosis^109^.

The competing endogenous RNAs (ceRNAs), such as long non-coding RNAs (lncRNA) and circRNAs, influence the post-transcriptional regulation of miRNAs through miRNA response elements^110^-^112^, ultimately modulating the functions of TAMs and CD8^+^ T cells^113^, ^114^. M2 TAMs-derived exosomes contain LINC01592, which can be transported into tumor cells and subsequently facilitate tumor growth by inhibiting the E2F6/NBR1/MHC-I signaling pathway. Consequently, inhibiting LINC01592 increases the MHC-I expression on the surface of tumor cells, thereby augmenting the efficacy of CD8^+^ T cell reinfusion therapy against tumors^113^. M2 TAMs exhibit high expression of circRNA MERTK, which mechanistically upregulates IL-10 expression in macrophages by sponging miR-125a-3p. This process leads to increased apoptosis of CD8^+^ T cells and fosters an immunosuppressive TME^114^.

## 6. Strategies for Repolarizing M2 TAMs to M1 Phenotypes

**Figure 4:**
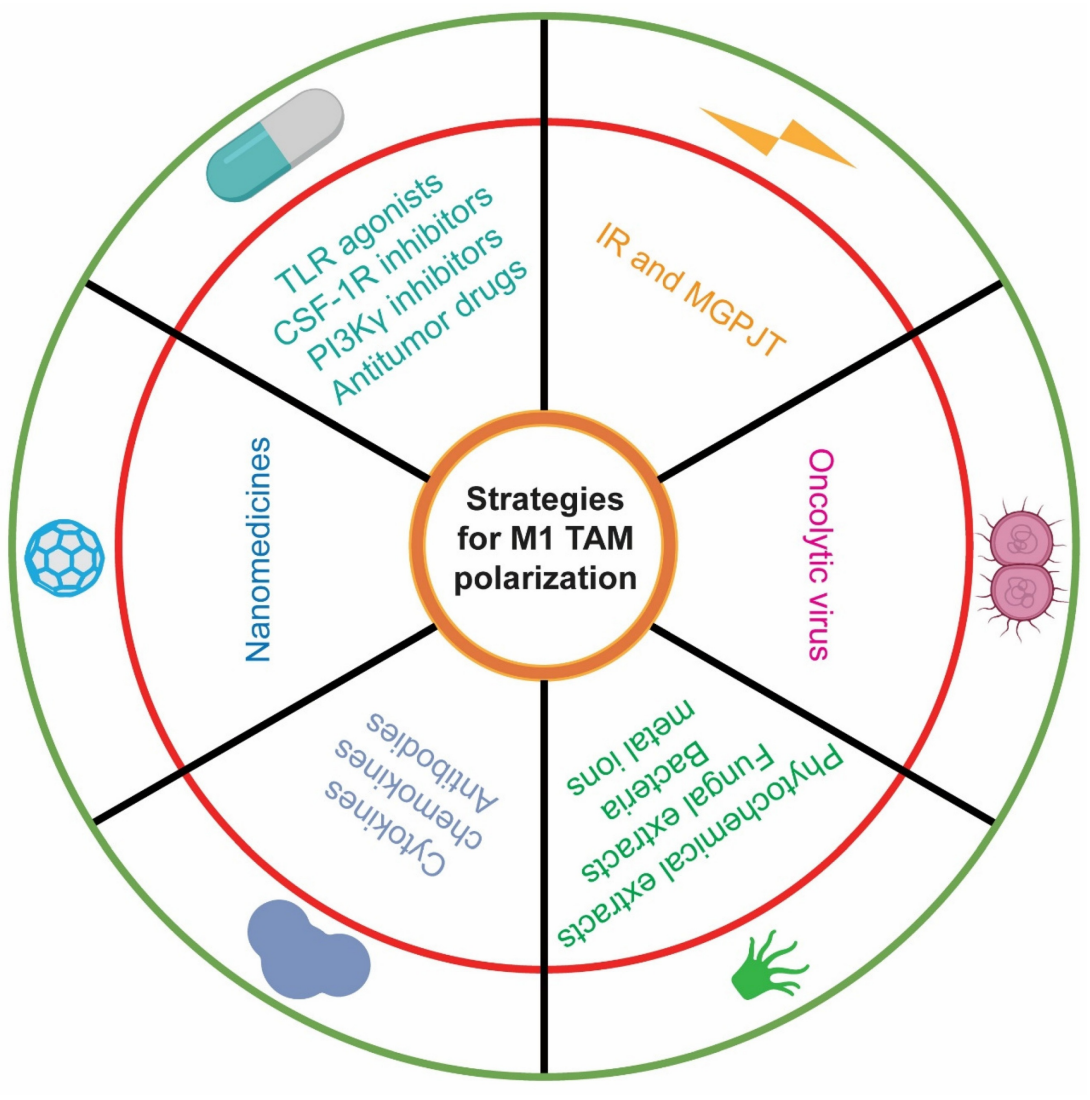
** Strategies for M1 TAM polarization.** Multifaceted treatment avenues have confirmed their potential to drive M1 tumor-associated macrophage (TAM) polarization, including small-molecule drugs, nanomedicines, molecular therapies, natural products, oncolytic viruses, and ionizing radiation. Specifically, Toll-like receptor (TLR) agonists, colony-stimulating factor-1 receptor (CSF-1R) inhibitors, and phosphoinositide 3-kinase γ (PI3Kγ) inhibitors are representative small-molecule drugs. Nanomedicines refer to the use of nanomaterials to deliver drugs, specific antibodies, and even gene-editing plasmids to reshape the TAM phenotype. Many proinflammatory cytokines and chemokines, as well as immune checkpoint blockade antibodies, constitute the mainstay of molecular therapy. Natural products comprise of phytochemical and fungal extracts, bacteria, and metal ions.

The M1/M2 ratio elucidates the immune architecture of the TME, with M1 TAMs being indicative of favorable patient prognoses, while M2 TAMs are emblematic of poor clinical outcomes^115^. Current strategies for TAM-related cancer therapy primarily encompass (1) the direct eradication of M2 TAMs, (2) the reduction of M2 TAM recruitment, and (3) the repolarization of M2 TAMs to the M1 phenotype. These modalities can effectively enhance CD8⁺ T antitumor immune responses and improve patient survival. By modulating the activity of the aforementioned signaling pathways, an increasing body of therapeutic paradigms are harnessed to repolarize TAM phenotypes and further potentiate the antitumor efficacy of CD8^+^ T cells **(Table 3; Figure 4)**.

### 6.1. Small-molecular drugs

TLR agonists trigger innate immune responses in macrophages and can reprogram M2 TAMs into M1 phenotypes, thereby diminishing Treg populations and promoting antigen-specific CD8^+^ T cell activation^116^-^118^. Additionally, they can attenuate PD-L1 expression in TAMs^116^ and facilitate the secretion of proinflammatory cytokines by TAMs^118^. A TLR3-specific adjuvant, in conjunction with the VISTA-specific monoclonal antibody 13F3, markedly enhances the CD8^+^ T cell/Treg and M1/M2 ratios. Furthermore, this combination therapy upregulates the expression of immunostimulatory molecules while concurrently downregulating immunosuppressive molecules^119^. A folate-targeted TLR7 agonist selectively delivers the drug to folate receptor-β positive macrophages in vivo, facilitating TAM polarization towards the M1 phenotype and enhancing CD8^+^ T cell infiltration. This synthetic compound significantly extends mouse survival without evident toxicity^120^.

The CSF-1/CSF-1R axis is crucial for the differentiation, proliferation, and survival of M2 TAMs^121^, ^122^. The CSF1R fosters an immunosuppressive TME by increasing the intratumoral infiltration of M2 TAMs and MDSCs. Consequently, CSF-1R inhibition emerges as a promising strategy to specifically target M2 TAMs and counteract tumor immune evasion^123^, ^124^. The selective CSF1R inhibitors remodels the TME into an immunostimulatory state by increasing M1 TAMs and CD8^+^ T cells while reducing M2 TAMs and MDSCs^125^, ^126^. Fusing IL-10 with a CSF-1R blockade antibody generates a bifunctional protein that effectively depletes TAMs and augments CD8^+^ T cell antitumor immunity, demonstrating significant antitumor activity across various solid tumors, particularly in head and neck cancers^127^.

As previously described, targeting the Mincle pathway is beneficial for reversing the polarization of M2 TAMs. Syk inhibitors can disrupt the Mincle signaling pathway^128^, ^129^. Aptamers, which are short, single-stranded DNA or RNA molecules, can bind to specific targets with high affinity. Mincle-specific aptamers have demonstrated therapeutic potential by selectively inhibiting Mincle activation^130^. Ultrasound-Mediated Bubble (USMB) technology enhances the delivery of therapeutic agents to specific tissues using ultrasound and microbubbles. It has shown promise in improving the efficiency and targeting of anti-Mincle agents^131^, ^132^. Potential therapeutic approaches targeting the Mincle pathway highlight their relevance in modulating TAM activity and enhancing antitumor immunity.

PI3Kγ inhibition can repolarize M2 TAMs to M1 phenotypes, thereby triggering an antitumor immune response^76^, ^133^, ^134^. The PI3Kγ inhibitors stimulates M1 TAM activation and antigen-presentation and augments the CD8^+^ T/Treg and M1/M2 ratios, while reducing IL-10 secretion by M2 TAMs. As a monotherapy, it further enhances the CD8^+^ T cell activation and boosts the ICB antitumor activity^135^, ^136^.

A panel of canonical (e.g., Vinblastine and Doxorubicin^137^, ^138^) and non-canonical antitumor drugs (e.g., Hydroxychloroquine, Verteporfin, and Sulfasalazine^139^-^141^) can remodel the TME by immunostimulating the functions of TAMs and CD8^+^ T cells. Consequently, their combination with PD-1/PD-L1 ICB therapy reconfigures the TME by increasing the CD8^+^ T cell/Treg and M1/M2 ratios, contributing to the robust antitumor responses^142^-^144^.

### 6.2. Nanomedicines

Given the substantial advantages of nanomaterials in cancer therapy, such as enhanced targeting precision, superior drug stability and bioavailability, and the augmented permeability and retention effect, researchers are endeavoring to design an array of nanomedicines to reprogram TAM phenotypes and augment CD8^+^ T cell functionality. Many nanomedicines repolarize M2 TAMs to M1 phenotype via activating NF-κB and TLR signaling pathways^145^-^150^, or obstructing STAT3 signaling pathway^151^, thereby reactivating CD8^+^ T cell-mediated immune responses.

Liposomes and lipid-coated calcium phosphate represent the promising antigen delivery systems and have been validated as an efficacious platform for vaccinations and drugs^152^-^156^. For example, Paclitaxel induces the polarization of M1 TAMs to M2 phenotypes and diminishes the expression of CXCL9/10 on macrophages^157^. Conversely, the concomitant delivery of Paclitaxel and Cryptotanshinone via liposomes inhibits STAT3 activation, thereby reversing the immunosuppressive TME^155^. CD169^+^ macrophages are situated in the marginal zone of the spleen and the subcapsular sinus of lymph nodes. GM3-αGC-OVA liposomes increase CD8^+^ T cell responses, which is closely related to CD169^+^ macrophages and the CD169 receptor^158^. A liposomal platform targeting CD169 for selective delivery to macrophages can enhance CD8^+^ T cell proliferation and regulate the activation ratio of CD4^+^/CD8^+^ T cells in vivo^159^, ^160^.

PD-L1 is an emerging target for TAM-directed therapies. Delivering siPD-L1 to M2 TAMs via nanocarriers facilitates their reprogramming to M1 TAMs^161^. The antitumor efficacy of the nano-PD-L1 trap surpasses that of PD-L1 monoclonal antibodies. Unlike PD-L1 monoclonal antibodies, the nano-PD-L1 trap can sustainably reduce the intratumoral accumulation of M2 TAMs and MDSCs^162^. Activation of Wnt/β-catenin signaling in antigen-presenting cells such as DCs and macrophages preferentially primes Tregs over CD8^+^ T cells. Utilizing β-catenin-targeting nanocarriers significantly upregulates CD80 and CD86 expression on macrophages while inhibiting CD206 and PD-L1 expression. Consequently, in an in vitro co-culture system, these macrophages can enhance CD8^+^ T cell proliferation^163^.

### 6.3. Molecular Therapies

Proinflammatory cytokines and chemokines are extensively utilized to modulate TAMs and CD8^+^ T cells within the TME. Some vaccine adjuvants enhance the antitumor immune response of TAMs and CD8^+^ T cells by activating TLR3^164^ and TLR4^165^. Type I and Type II IFNs can induce macrophage polarization towards the M1 phenotype, augments their phagocytic activity and the production of proinflammatory cytokines^166^-^169^. The combination of anti-CD20 and a mutated IL-2 (no-alpha mutein) promotes the release of proinflammatory cytokines by CD8^+^ T cells and the expression of immunostimulatory molecules on the surface of TAMs^170^.

Dual-targeting ICB therapy effectively reverses the immunosuppressive ecosystem via stimulating TAM functionality. Dual-targeting CD47/PD-L1 ICB therapy or combined with FOLFOX strategy markedly increases the M1/M2 ratio and activated CD8^+^ T cell populations in the TME^171^, ^172^. Dual-targeting PD-L1/CTLA4 antibody plus TGF-β inhibitor significantly increases M1 TAM populations^173^. However, dual-targeting PD-1/CTLA-4 ICB therapy-amplified CD8^+^ T cell infiltration and antitumor response are weakened by depleting CXCL9 expression on TAMs^174^.

Targeting specific biomarkers on M2 TAMs has shown potential to reprogram them to the M1 subtype, thereby enhancing CD8^+^ T cell responses. MARCO is predominantly expressed on macrophages, particularly M2 TAMs, where it enhances IL-10 production and Treg proliferation^175^. Targeting MARCO on TAMs with antibody can re-educate them towards the M1 subtype^176^. Chimeric antigen receptor macrophages (CAR-Ms) dual-targeting HER2/CD47 phagocytose antigen-specific tumor cells. These engineered CAR-Ms also reprogram M1 TAM polarization and stimulate CD8^+^ T cells to secrete a plethora of proinflammatory molecules^177^.

### 6.4. Natural Products

Many phytochemical extracts in nature, renowned for their anti-inflammatory and antioxidant properties, modulate TAMs and CD8^+^ T cells within the TME. Several traditional Chinese medicines (e.g., Compound Kushen Injection, Resveratrol, and *Terminalia bellirica*) drives the polarization of M2 TAMs to M1 phenotypes and augments the activation of intratumoral CD8^+^ T cells, ultimately leading to the shrinkage of solid tumors^178^-^180^. Moreover, Naringenin and Phytohemagglutinin potentiate CD8^+^ T cell activation by stimulating macrophages in the TME, likely through improved antigen processing and presentation^181^, ^182^. Cannabigerol can reduce the secretion of CSF-1 in solid tumors, such as melanoma, thereby remodeling M1 TAMs and reinstating CD8^+^ T cell activation^183^.

Fungal extracts exhibit potent efficacy in educating M1 TAM polarization and invigorating CD8^+^ T cell antitumor immunity^184^, ^185^. L-ergothioneine lacks intrinsic immunostimulatory properties but can potentiate TLR responses in macrophages, thereby eliciting robust innate immune activity^185^. When conjugated with a TLR2-containing cancer vaccine, it markedly enhances M1 TAM infiltration and CD8^+^ T cell activation^186^.

A variety of bacteria (e.g., *Streptococcus salivarius* and Bacillus Calmette-Guérin lysate) and metal ions (e.g., Mn²⁺) both polarize TAMs to M1 phenotype and augments their maturation and antigen presentation, thus inducing memory CD8^+^ T cell amplification, and CD8^+^ T cell activation^187^-^189^. Conversely, the microbiome in PDAC patients creates an immunosuppressive TME by differentially activating specific TLRs in macrophages. However, bacterial ablation can immunostimulate the tumor immune microenvironment and enhance the antitumor efficacy of PD-1 ICB therapy^190^.

### 6.5. Oncolytic Virus

Oncolytic viruses are a class of viruses that selectively target and eradicate tumor cells while sparing jeopardy to normal tissues. Additionally, they can activate TAMs and CD8^+^ T cells to further eradicate residual tumor cells. For example, CARG-2020 is a self-amplifying virus-like vesicle that encodes immune regulatory genes to modulate various immune signaling pathways. It educates M2 TAMs polarizing towards M1 phenotypes and augments CD8^+^ T cell responses^191^.

### 6.6. Ionizing radiation (IR) and medical gas plasma jet technology (MGPJT)

IR not only directly disrupts the DNA replication of tumor cells but also reconfigures the TME through influencing TAM phenotypes and CD8^+^ T cell activity. Valproic acid (VPA) and its derivative HPTA are potent immune activators for radiotherapy. The combination of VPA/HPTA and radiotherapy can induce the polarization of TAMs towards the M1 phenotype, increase the number of activated CD8^+^ T cells and elicit a substantial production of inflammatory cytokines during the early stage of treatment^192^. Conversely, two weeks of radiotherapy increases STAT3-activated TAMs and reduces CD8^+^ T cells. Targeting local TLR9/STAT3 signaling promotes the local accumulation of M1 TAMs and CD8^+^ T cells^193^. Notably, targeting local STAT3 is challenging because, although STAT3 diminishes CD8^+^ T cell cytotoxicity and enhances Treg tolerance, it is indispensable for the expansion of memory T cells and long-term tumor immunity^194^-^197^. ATM inhibition intensifies IR-induced DNA damage, which upregulates type I IFN expression in macrophages through the STING/IRF3 signaling pathway, thereby augmenting IR-induced CD8^+^ T cell antitumor immunity^198^.

MGPJT is an innovative medical treatment method that harnesses plasma, often referred to as the fourth state of matter. This technology involves ionizing a gas using radio frequency or microwave energy to generate a plasma jet, which produces reactive oxygen and nitrogen species (ROS and RNS)^199^. These ROS and RNS can induce immunogenic cell death in tumor cells, thereby activating the immune system and polarize M2 TAM to M1 phenotype by releasing tumor antigens^200^. By modulating oxidative stress levels and cytokine profiles within the TME, gas plasma jets can potentiate the antitumor immune response of TAMs^199^, ^200^. The plasma jet-generated ROS and RNS can directly eradicate tumor cells, leading to the release of DAMPs^201^. These DAMPs can further stimulate the immune system and reprogram TAMs to bolster their antitumor activities^201^, ^202^. Consequently, MGPJT represents a promising approach to harnessing the body's intrinsic defenses against cancer.

## 7. Conclusions and Perspectives

We have posed and contextualized the tumor cell-macrophage-CD8^+^ T cell loop where macrophage is prone to be reprogrammed. Recent works that delve into the intricate mechanisms underlying TAM immunostimulatory and immunosuppressive polarization have been further explored. These mechanisms inform and enhance innovative or combinatory therapeutic strategies aimed at modulating TAMs towards the M1 phenotype. Such novel interventions can substantially ameliorate the immunosuppressive milieu of the TME, either as standalone treatments or in combination with ICB therapy. Emerging technologies, like single-cell RNA sequencing and spatial transcriptomics, are unraveling the functional diversity of TAMs and their crosstalk with CD8^+^ T cells. These insights provide a rational basis for optimizing combinatorial strategies that integrate TAM reprogramming with ICB therapy.

Future endeavors should expeditiously translate strategies for immunostimulatory reprogramming TAM phenotypes into tangible clinical benefits for cancer patients. Our advanced understanding of TAM polarization paves the way for the development of cutting-edge therapeutic approaches that functionally immunostimulate TAMs and subsequently augment CD8^+^ T cell antitumor immunity, potentially providing many promising avenues to better optimize clinical benefits for cancer patients undergoing ICB treatment.

## Figures and Tables

**Table 1:** The correlation of macrophages and CD8^+^ T cells in different cancer types.

Cancer subtype	Macrophage status	T cell status	Refs
MICAL2 in pancreatic cancer	Increased presence of M2 TAMs	Decreased presence of CD8^+^ T cells	17
YBX1+ luminal breast cancer	Increased presence of M2 TAMs	Increased T cell exhaustion	18
GM-CSFRα+ Intrahepatic cholangiocarcinoma	Decreased presence of M2 TAMs	Increased presence of CD8^+^ T cells	19
KCTD5+ lung adenocarcinoma	Increased presence of TAMs	Increased presence of CD8^+^ T cells	20
ARHGAP39+ breast cancer	Decreased presence of TAMs	Decreased presence of CD8^+^ T cells	21
OPN- glioma	Decreased presence of M2 TAMs	Increased CD8^+^ T cells cytotoxicity	22
MTA1+ Colon Cancer	Increased presence of TAMs	Decreased CD8^+^ T cells cytotoxicity	23
MISP+ hepatocellular carcinoma	Decreased presence of M2 TAMs	Increased presence of CD8^+^ T cells	24
GPC3+ ovarian cancer	Increased presence of M1 TAMs	Increased CD8^+^ T cells cytotoxicity	25

**Table 2:** Potential signaling pathways for reprogramming tumor-associated macrophages.

M1 TAM polarization	M2 TAM polarization
NF-κB signaling activation^35-38^	NF-κB signaling inactivation^51-53^
STAT1/4 signaling activation^40-42^	STAT3/6 signaling activation^57, 59, 60, 68-71^
STING signaling activation^43, 44^	Mincle pathway activation^73, 74^
Notch1/2 signaling activation^47^	Syk-PI3K signaling activation^75-80^
PRR signaling activation^48, 49^	

**Table 3:** The detailed avenues for M1 TAM polarization.

Classifications	Subclassifications	Treatment strategies	Effects	Study status	Refs
Small-molecular drugs	TLR agonists	TLR3 Agonist	Enhanced TAM antigen presentation, and increased CD8^+^ T cell activation	Preclinical	116
		SMU-L11 (TLR7 agonist)	M1 TAM polarization, and increased CD8^+^ T cell proliferation and activation	Preclinical	118
		Folate-targeted TLR7 agonist	Increased M1/M2 ratio, and increased CD8^+^ T cell infiltration	Preclinical	120
	CSF1R kinase inhibitor	Q702	Increased M1/M2 ratio, and increased CD8^+^ T cell populations	Preclinical	125
	Inhibitor of FGFR1/2/3 and CSF-1R	3D185	Increased M1/M2 ratio, and increased CD8^+^ T cell activation	Preclinical	126
	Targeting the Mincle pathway	USMB-shMincle	M1 TAM polarization	Preclinical	132
	PI3Kγ inhibitor	AZD3458	Enhanced TAM activation, and increased CD8^+^ T cell antitumor activity	Preclinical	135
	Pan-PI3K inhibitor	Copanlisib	Enhanced CD8^+^ T cell/Treg and M1/M2 ratios, and increased infiltration of CD8^+^ T cells and TAMs	Preclinical	136
Nanomedicines	TLR7/8 agonist	R848@LNPs	Increased M1 TAM and CD8^+^ T cell populations	Preclinical	145
	TLR3 agonist, NF-κB activation	FP-NPs	M1 TAM polarization	Preclinical	146
	NF-κB activation	Ferumoxytol	Increased M1 TAM populations	Preclinical	147
	TLR7/8 agonist	Telratolimod	Increased M1 TAM populations, and increased CD8^+^ T cell antitumor activity	Preclinical	148
	TLR9 agonist	LCpG	M1 TAM polarization, and increased CD8^+^ T cell proliferation	Preclinical	149
	TLR7 agonist	T7-Exo/siGalectin-9	M1 TAM polarization	Preclinical	150
	STAT3 signaling blockade	CpGgel-siSTAT3	Enhanced M1 TAM activation, and increased infiltration of CD8^+^ T cells	Preclinical	151
	STAT3 signaling blockade	CS/LyP-1-PC	Decreased infiltration of Tregs, M2 TAMs, and MDSCs	Preclinical	155
	Targeting splenic CD169^+^ macrophages	GM3-αGC-OVA	Increased CD8^+^ T cell antitumor immunity	Preclinical	158, 160
	Delivering siPDL1 into M2 TAMs	7D12-mExo-M2pep-siPDL1	M1 TAM polarization, and increased CD8^+^ T cell antitumor immunity	Preclinical	161
	Nanodelivery of PD-L1 expression in M2 TAMs	nano-PD-L1 trap	Reduced M2 TAM proportion, and increased CD8^+^ T cell activation	Preclinical	162
	Targeting Wnt/β-catenin signaling	XAV-Np	Increased M1/M2 ratio, and increased CD8^+^ T cell proliferation	Preclinical	163
Molecular therapy	TLR3 agonist	Poly-ICLC	M1 TAM polarization	Preclinical	164
	TLR4 agonist	Monophosphoryl lipid A	M1 TAM polarization, and increased CD^+^ 8 T cell activation	Preclinical	165
		Type I IFN	Increased CD8^+^ T cell activation	Preclinical	168
		IFN-γ	Increased infiltration of M1 TAMs and CD8^+^ T cells	Preclinical	169
		Dual-targeting CD47/PD-L1 antibody	Increased CD8^+^ T cell activation, and increased infiltration of M1 TAMs	Preclinical	171
		Combination of dual-targeting CD47/PD-L1 antibody and FOLFOX	Decreased infiltration of Tregs and MDSCs, increased M1/M2 ratio, and increased CD8^+^ T cell activation	Preclinical	172
		Combination of dual-targeting PD-L1/CTLA4 antibody and TGF-β inhibitor	Increased infiltration of M1 TAMs	Preclinical	173
		Dual-targeting HER2/CD47 CAR-Ms	Shift of TAM phenotype, and increased CD8^+^ T cell activation	Preclinical	177
Natural products	Chinese medicines	Compound Kushen Injection	Increased CD8^+^ T cell proliferation and activation	Preclinical	178
		Resveratrol	Reduced proportion of M2 TAMs, and increased CD8^+^ T cell activation	Preclinical	179
		Terminalia bellirica	M1 TAM polarization, and increased CD8^+^ T cell antitumor immunity	Preclinical	180
		Naringenin	Lymph node CD169^+^ macrophage activation and increased infiltration of CD8^+^ T cells	Preclinical	181
		phytohemagglutinin	Increased CD8^+^ T cell proliferation	Preclinical	182
		Cannabigerol	Remodeling M1 TAMs, and increased CD8^+^ T cell activation	Preclinical	183
	Fungal extracts	Lentinan	Enhanced TAM activation, and increased CD8^+^ T cell proliferation	Preclinical	184
		L-ergothioneine	Increased infiltration of M1 TAMs and increased CD8^+^ T cell activation	Preclinical	186
	Bacteria	*Streptococcus salivarius*	M1 TAM polarization and enhanced antigen presentation, and increased CD8^+^ T cell proliferation and activation	Preclinical	187
		BCG hydrogel	M1 TAM polarization and enhanced antigen presentation	Preclinical + clinical	188
	Metal ions	Manganese	Enhanced M1 TAM maturation and antigen presentation, increased CD8^+^ T cell differentiation and activation	Clinical	189
Oncolytic virus		CARG-2020	Increased M1 TAM differentiation, decreased MDSC expansion, and increased CD8^+^ T cell antitumor immunity	Preclinical	191
IR and MGPJT	IR	Combination of VPA/HPTA and radiotherapy	M1 TAM polarization, and increased CD8^+^ T cell activation	Preclinical	192
	MGPJT	MGPJT	M1 TAM polarization	Preclinical	199

This table is related to Figure 4, which provides all strategies for M1 TAM polarization.
